# Regulated cell death pathways and their roles in homeostasis, infection, inflammation, and tumorigenesis

**DOI:** 10.1038/s12276-023-01069-y

**Published:** 2023-08-23

**Authors:** Ein Lee, Chang-Hyun Song, Sung-Jin Bae, Ki-Tae Ha, Rajendra Karki

**Affiliations:** 1 Department of Biomedical Sciences, College of Medicine, Seoul National University, Seoul, 03080 South Korea; 2 Department of Biological Sciences, College of Natural Science, Seoul National University, Seoul, 08826 South Korea; 3 Department of Molecular Biology and Immunology, College of Medicine, Kosin University, Busan, 49267 South Korea; 4 Department of Korean Medical Science, School of Korean Medicine, Pusan National University, Yangsan, 50612 South Korea; 5 Nexus Institute of Research and Innovation (NIRI), Kathmandu, Nepal

**Keywords:** Cell death and immune response, Stress signalling

## Abstract

Pyroptosis, apoptosis, necroptosis, and ferroptosis, which are the most well-studied regulated cell death (RCD) pathways, contribute to the clearance of infected or potentially neoplastic cells, highlighting their importance in homeostasis, host defense against pathogens, cancer, and a wide range of other pathologies. Although these four RCD pathways employ distinct molecular and cellular processes, emerging genetic and biochemical studies have suggested remarkable flexibility and crosstalk among them. The crosstalk among pyroptosis, apoptosis and necroptosis pathways is more evident in cellular responses to infection, which has led to the conceptualization of PANoptosis. In this review, we provide a brief overview of the molecular mechanisms of pyroptosis, apoptosis, necroptosis, and ferroptosis and their importance in maintaining homeostasis. We discuss the intricate crosstalk among these RCD pathways and the current evidence supporting PANoptosis, focusing on infectious diseases and cancer. Understanding the fundamental processes of various cell death pathways is crucial to inform the development of new therapeutics against many diseases, including infection, sterile inflammation, and cancer.

## Cell death: Interactions between different pathways

The different pathways that cause cell death, which are activated under different conditions, have complex relationships and sometimes act together to fight infection or cancer. All the pathways eliminate cells damaged by disease or mutation and are important for development and disease resistance, but can affect immune responses to infection or cause damage if dysregulated. Rajendra Karki at Seoul National University in South Korea and co-workers review recent evidence revealing links, including common molecules, between the pathways. Because cancer cells can become resistant to anti-cancer drugs or to the usual closely regulated cell death pathway, the ability to trigger a different type of cell death or to activate all pathways holds great promise for cancer treatment. Improving our understanding of cell death could help in treating multiple diseases, from infection to cancer.

## Introduction

**Fig. 1:**
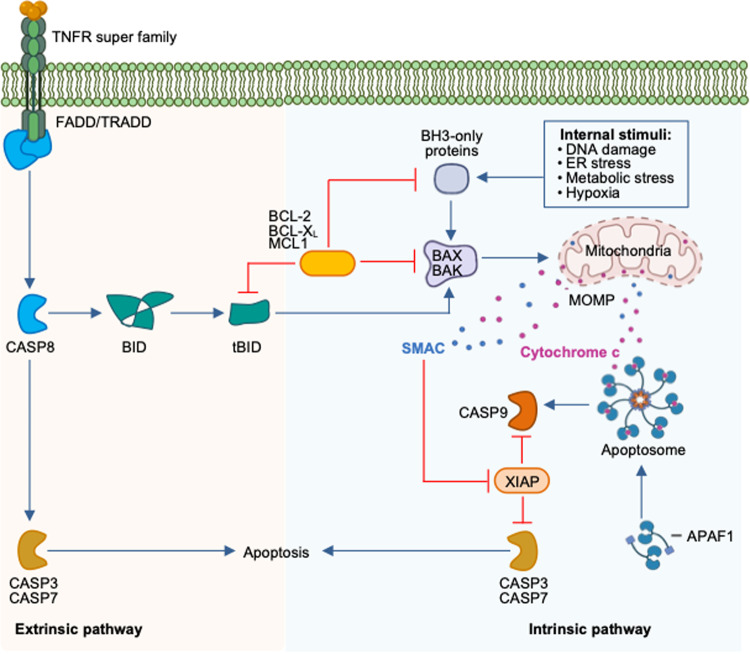
Molecular mechanisms of extrinsic and intrinsic apoptosis. Extrinsic apoptosis: Binding of a ligand such as FASL, TNF, TRAIL, and TWEAK to one of several death receptors (TNF receptor superfamily) initiates extrinsic apoptosis by triggering receptor oligomerization and the recruitment of adaptor proteins containing death domains such as TRADD and FADD. The resulting complexes activate caspase-8, which activates executioners caspase-3 and caspase-7. Intrinsic apoptosis: Diverse cytotoxic stimuli, such as DNA damaging agents and stress, activate BH3-only family members, thereby activating pro-apoptotic effectors BAX and BAK, which then disrupt the mitochondrial outer membrane. The cytochrome c released from the mitochondria interacts with APAF1 to form apoptosomes, which in turn activate the initiator caspase-9. Crosstalk between the extrinsic and intrinsic pathways can occur through BID cleavage by caspase-8, leading to activation of BAX and BAK. The two pathways converge at activation of the effector caspases (caspase-3 and caspase-7).

**Fig. 2:**
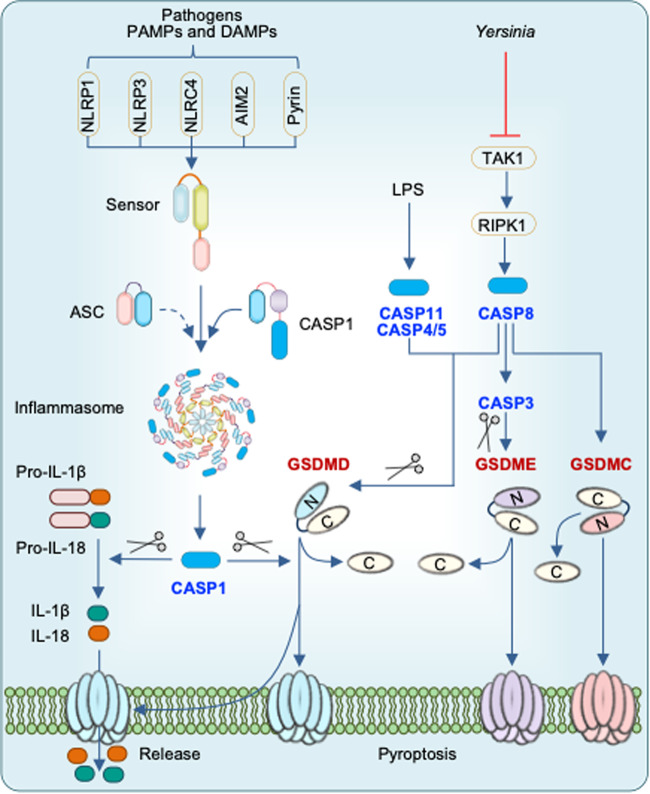
Inflammasome activation and pyroptosis. Certain pathogens, PAMPs, and DAMPs are sensed by specific sensors to assemble an inflammasome consisting of a sensor, ASC, and caspase-1. Active caspase-1 cleaves pro-IL-18 and pro-IL-1β into their mature forms. Active caspase-1, caspase-11, and caspase-8 cleave GSDMD to free the N-terminal region, which undergoes oligomerization to form pores in the plasma membrane. Active caspase-8 also cleaves GSDME and GSDMC. Pore formation in the plasma membrane by GSDMs causes cell lysis and release of intracellular contents and the inflammatory cytokines IL-18 and IL-1β following their maturation by caspase-1.

**Fig. 3:**
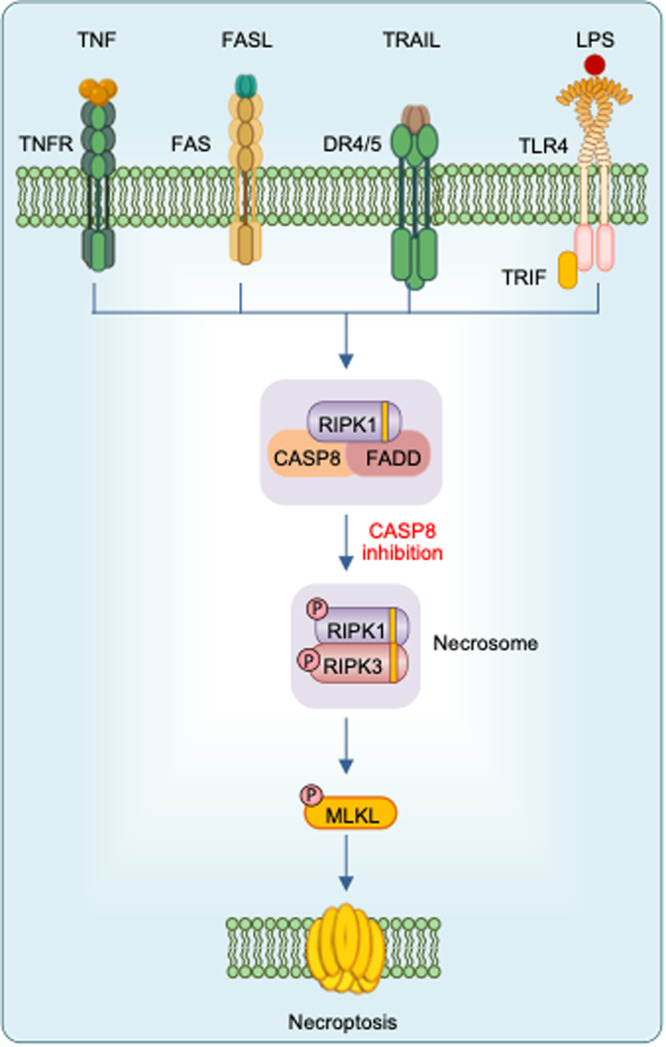
Molecular mechanisms of necroptosis. TNF ligation or LPS stimulation results in activation of NF-κB signaling. Inactivation of NF-κB signaling or engagement of death receptors triggers the assembly of an apoptosis-inducing complex consisting of FADD, caspase-8, and RIPK1. When caspase-8 is inhibited, RIPK1 and RIPK3 form necrosomes through homotypic interactions with RHIM, resulting in phosphorylation of MLKL. Phosphorylated MLKL undergoes oligomerization and induces membrane rupture.

**Fig. 4:**
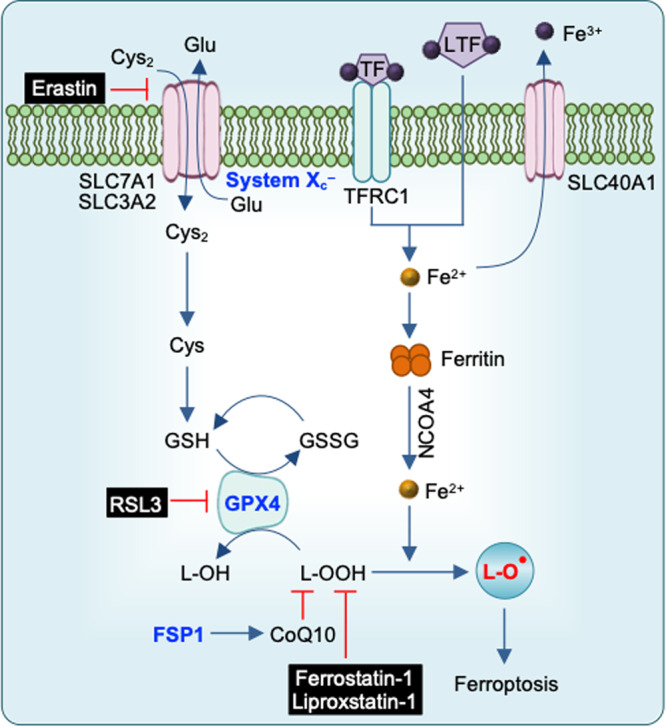
Molecular mechanisms of ferroptosis. Ferroptosis is primarily driven by iron-dependent lipid peroxidation. Iron bound to transferrin is transported into cells by TFRC1. NCOA4-mediated ferritinophagy increases the free iron pool. Ferroptosis is inhibited by GSH, the synthesis of which involves the uptake of cystine via the cystine-glutamate antiporter (system X_c_^-^). Using GSH as a cofactor, GPX4 reduces phospholipid hydroperoxides to their corresponding alcohols. The FSP1-CoQ10 system inhibits ferroptosis.

Cell death is a conserved fundamental process that plays a central role in all aspects of life. It is involved in embryonic development, maintaining organismal homeostasis, and eliminating damaged cells. Cell death can be induced in response to physical damage and infection^1,2^. Based on their signal dependency, cell death can be classified into regulated or nonregulated cell death. While regulated cell death (RCD) is tightly regulated by intracellular signal transduction pathways, non-RCD is accidental and results from unexpected cell injury. Considering the morphological characteristics and molecular mechanisms, RCD can be further classified as nonlytic and lytic cell death^3^. Apoptosis is a nonlytic form of cell death in which the cell retains membrane integrity and exhibits cytoplasmic shrinkage, chromatin condensation, nuclear fragmentation, and plasma membrane blebbing (Table 1). In contrast, pyroptosis, necroptosis, and ferroptosis are classical lytic cell death processes, which result in the removal of dead cells and the release of potent inflammatory mediators (Table 1). Therefore, apoptosis is typically ‘immunologically silent’, whereas pyroptosis, necroptosis, and ferroptosis are referred to as relatively ‘violent’ types of cell death^3^. There are other RCD, including parthanatos, lysosome-dependent cell death, autophagy-dependent cell death, alkaliptosis, oxeiptosis, and cuproptosis^4,5^. As the field continues to progress, several molecules that regulate cell death have been identified that establish cell death as a regulated process. Although work over the past three decades has identified several distinct types of RCD, the molecular mechanisms responsible for the initiation, transduction, and execution of pyroptosis, apoptosis, necroptosis, and ferroptosis are the most well established. Molecularly, apoptosis is executed by activation of the executioner caspases caspase-3 (CASP3) and CASP7 downstream of the initiator caspases CASP8, CASP9, and CASP10^6–8^ (Fig. 1). Plasma membrane pore formation by activated gasdermin family members such as GSDMD or GSDME leads to pyroptosis. Inflammatory caspases, CASP1 and CASP11 (mice) or CASP4/5 (humans) activate GSDMD^9,10^ (Fig. 2). Necroptosis is driven by the formation of mixed lineage kinase domain-like pseudokinase (MLKL) pores following MLKL phosphorylation downstream of the receptor-interacting protein kinase 1 (RIPK1) and RIPK3 signaling axis^11,12^ (Fig. 3). Although ferroptosis is usually accompanied by a large amount of iron accumulation and lipid peroxidation^13^, the molecules involved in the execution of ferroptosis are not known (Fig. 4). Plasma membrane rupture mediated by ninjurin 1 (NINJ1) is required for the release of larger DAMPs such as LDH and HMGB1 during lytic cell death^14^.

**Table 1:** Morphological features of different types of regulated cell death.

Regulated cell death (RCD)	Morphological Features									
	Lysis	Membrane rupture	Pore formation	Cell swelling	Organelle swelling	Cell shrinkage	Membrane blebbing	Chromatin condensation	DNA damage	Intact nucleus
Pyroptosis	**√**	**√**	**√**	**√**	Χ	Χ	**√**	**√**	**√**	**√**
Apoptosis	Χ	Χ	Χ	Χ	Χ	**√**	**√**	**√**	**√**	Χ
Necroptosis	**√**	**√**	**√**	**√**	**√**	Χ	Χ	Χ	**√**	**√**
PANoptosis	**√**	**√**	**√**	**√**	**√**	**√**	**√**	**√**	**√**	**√**

The table shows the morphological features of pyroptosis, apoptosis, necroptosis, and PANoptosis.

The identification of key regulators of pyroptosis, apoptosis, necroptosis, and ferroptosis has increased the understanding of cell death functions in multiple settings ranging from organismal homeostasis to infectious, inflammatory, and autoimmune diseases and cancer. While inflammatory cell death—pyroptosis, necroptosis, and ferroptosis—is involved in providing host defense against invading pathogens, apoptosis ensures normal development and cellular homeostasis^1,2^. For instance, mice with defective apoptosis, such as those with mutations in or lacking apoptotic regulators such as caspase-9, APAF1, BAK, BAX, and BOK, typically die during late stages of development or soon after birth^15–17^. Conversely, despite the impaired responses to certain pathogens or other external insults, mice deficient in pyroptosis, such as those lacking GSDMD and GSDME, or necroptosis, such as those lacking RIPK3 and MLKL, are born healthy^9–12^.

**Fig. 5:**
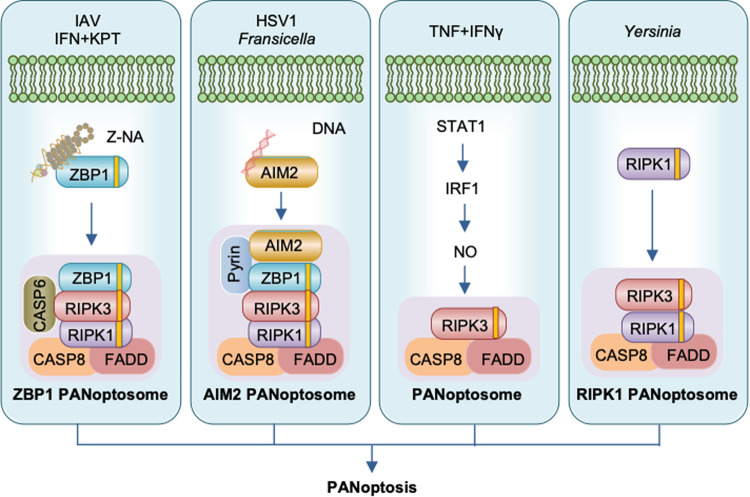
Triggers and molecules involved in PANoptosome assembly. Pathogens such as IAV, HSV1, *Francisella,* and *Yersinia* and other agents such as IFN + KPT and TNF + IFNγ have been identified to induce PANoptosis. ZBP1 senses IAV or endogenous Z-NA to assemble ZBP1 PANoptosomes consisting of ZBP1 and other cell death molecules. CASP6 potentiates the interaction between RIPK3 and ZBP1. AIM2 senses dsDNA during HSV1 or *Francisella* infection to assemble the AIM2 PANoptosome. TNF + IFNγ activates STAT1 to induce IRF1-dependent NO release, which activates CASP8 and RIPK3 to trigger PANoptosis. During *Yersinia* infection, RIPK1 assembles the RIPK1 PANoptosome.

Although apoptosis, necroptosis, and pyroptosis have historically been considered independent, there is now mounting evidence that these RCD pathways are interconnected at multiple levels. Moreover, activation of biochemical markers from all three RCD pathways has been observed with several sterile triggers, such as the combination of interferon (IFN) and nuclear export inhibitors (NEI), the combination of TNF and IFN-γ and TAK1 inhibitors and nonsterile triggers, such as bacterial and viral infection^1^. The combined loss of these RCD pathways, but not individual RCD pathways, prevents the cell death induced by these triggers implying a united modality of death defined as PANoptosis (Fig. 5). The pathophysiological relevance of PANoptosis has been observed during infections as well as in autoinflammatory diseases, cytokine storms and cancer^18–23^.

In this review, we will provide a framework for understanding the different types of RCD pathways. We will discuss the integral components of pyroptosis, apoptosis, necroptosis, and ferroptosis and summarize the latest insights into molecular and functional connections among the different RCD pathways that have led to the conceptualization of PANoptosis. In addition, we will summarize the pathological contexts involving these RCD pathways and emerging therapeutic applications of modulating them. Finally, we will explain the concept of PANoptosis that will likely drive the next decade of cell death studies.

## Regulated cell death

### Apoptosis

Apoptosis was the first RCD to be described and is the most studied form of cell death^24^. It is primarily associated with development and homeostasis. Cell shrinkage and pyknosis are the characteristic features of apoptosis (Table 1). Extensive plasma membrane blebbing follows the formation of apoptotic bodies consisting of cytoplasm with tightly packed organelles. These bodies are subsequently phagocytosed by surrounding cells such as macrophages and parenchymal cells and degraded within phagolysosomes, thus likely preventing secondary necrosis. Based on molecular events, there are two main apoptotic pathways – the intrinsic or mitochondrial pathway and the extrinsic or death receptor pathway (Fig. 1). The proteolytic cascade leading to activation of caspases is one of the biochemical features of both extrinsic and intrinsic apoptosis^2^. Intrinsic apoptosis can be induced by various agents that can trigger a variety of microenvironmental perturbations, including DNA damage, ER and replication stress, microtubular alterations, or mitotic defects. Mitochondrial outer membrane permeabilization (MOMP), which is controlled by proapoptotic and antiapoptotic members of the BCL2 family, is a critical step in intrinsic apoptosis^25^. In response to apoptotic stimuli, BAX and BAK form pores across the outer mitochondrial membrane (OMM) and possibly other intracellular membranes in association with other proapoptotic BH3-only proteins. MOMP is antagonized by antiapoptotic members of the BCL2 family, which directly bind to proapoptotic members. The regulation of apoptosis by BCL-2 family members is critical and has been extensively reviewed elsewhere^25^. MOMP promotes the cytosolic release of apoptogenic factors, including cytochrome c and SMAC, that normally reside in the mitochondrial intermembrane space^26^. The cytosolic pool of cytochrome c binds to APAF1 and pro-caspase 9 (CASP9) to form the supramolecular complex called the apoptosome, which activates CASP9^27^ (Fig. 1). Activated CASP9 is responsible for the activation of the downstream effector caspases CASP3 and CASP7. Extrinsic apoptosis is initiated through the engagement of two types of plasma membrane receptors: 1) death receptors, the activation of which depends on the binding of the cognate ligand, and 2) dependence receptors, which are activated upon dropping of their ligand level below a threshold^28^. Death receptors include Fas (CD95) and TNF receptor (TNFR). Upon ligand binding, a conformational change occurs that allows DD homotypic interactions between cytoplasmic adapter proteins such as FADD or TRADD and the receptors. The engagement of the Fas receptor results in the binding of FADD, whereas the binding of the TNF ligand to the TNF receptor engages TRADD with the recruitment of FADD and RIP. FADD then associates with pro-caspase-8 to form DISC, which leads to processing of caspase-8 to its active form^29,30^ (Fig. 1). The molecular mechanisms regulating CASP8 activity upon death receptor ligation have been extensively reviewed elsewhere^28^. Depending upon the cell type, the execution of extrinsic apoptosis follows two distinct pathways. The CASP8-dependent proteolytic activation of CASP3 and CASP7 is sufficient to execute cell death in type I cells such as thymocytes and mature lymphocytes^31^. However, in type II cells such as hepatocytes, pancreatic β cells and cancer cells, the activation of CASP3 and CASP7 is restrained by XIAP^32^. Therefore, type II cells require the proteolytic cleavage of BID by CASP8 to generate a truncated form of BID (tBID) that translocates to the OMM, leading to CASP9-driven cell death. Dependence receptors, which consist of 20 members, promote cell survival, proliferation, and differentiation when sufficient cognate ligands are available. However, these receptors activate the cell death cascade when ligand availability drops below a threshold value. For example, the dependence receptor DCC (deleted in colorectal cancer) promotes the activation of the CASP9-CASP3 cascade in the absence of its ligand^33,34^. Altogether, CASP9 and CASP8 are the initiator caspases of the intrinsic and extrinsic apoptosis pathways, respectively, and these pathways converge for activation of the same executioner enzymes: CASP3 and CASP7.

### Pyroptosis

Pyroptosis is a form of RCD that occurs in response to perturbations associated with innate immunity. The term pyroptosis was first coined by Cookson and Brennan to define apoptosis as RCD, which is dependent on inflammatory CASP1^35^. Depending on the initiating stimulus, pyroptosis is induced by inflammatory or apoptotic caspases, including CASP1, murine CASP11, human CASP4 and CASP5, and CASP3^36–39^. The assembly of an inflammasome, which includes a multiprotein complex containing a sensor, the adaptor ASC, and CASP1, leads to the autoprocessing of CASP1 into its active form. The well-established canonical inflammasome sensors include NLRP1, NLRP3, NLRC4, AIM2, and Pyrin^40^ (Fig. 2). The inflammasome adaptor protein ASC bridges inflammasome sensors and CASP1, leading to CASP1 activation. Active CASP1 cleaves its downstream substrates, including the inflammatory cytokines pro-IL-1β and pro-IL-18 to produce their bioactive forms and GSDMD to facilitate plasma membrane pore formation^40^. In addition, LPS detection by murine CASP11 or human CASP4/5 induces GSDMD cleavage by CASP11 to form membrane pores, which facilitate NLRP3 inflammasome activation in a cell-intrinsic manner to induce IL-1β and IL-18 maturation^36^. Therefore, pyroptosis is often associated with inflammasome activation and the release of cytokines, including IL-1β and IL-18, conferring robust proinflammatory effects. In addition to GSDMD, the N-terminal domains of other members of the gasdermin family, including GSDMA, GSDMB, GSDMC, and GSDME, can also induce pyroptosis in a context-dependent manner^39^ (Fig. 2).

### Necroptosis

Necroptosis is a form of the RCD pathway that is induced by specific death receptors, including Fas and TNFR1, or PRRs, such as TLR3 and TLR4, when caspase activation is inhibited (Fig. 3). The first genetic evidence of necroptosis was reported in T cells that underwent cell death in a FADD/RIPK1 manner without the release of cytochrome c^41^. Molecularly, necroptosis critically depends on the sequential activation of RIPK3 and MLKL^12^. In necroptosis initiated by TNFR1, the kinase activity of both RIPK1 and RIPK3 is essential for cell death, and RIPK1 and RIPK3 interact to form a necrosome through their RIP homotypic interaction motifs (RHIMs)^42^. Accordingly, chemical inhibitors of RIPK1, such as Nec-1, potently inhibit TNFR1-driven necroptosis^43^. Alternatively, RIPK3 can be activated following the RHIM-dependent interaction with TRIF upon either TLR3 or TLR4 activation^44^. Active RIPK3 phosphorylates MLKL, resulting in the formation of MLKL oligomers in the plasma membrane, which trigger plasma membrane permeabilization and cell death (Fig. 3). Considering that the inhibition of caspases, such as CASP8, is a prerequisite for necroptosis to occur, it is likely that necroptosis plays a “fail-safe” role in driving cell death in conditions that abrogate caspase activation^45,46^.

### Ferroptosis

Distinct from pyroptosis, apoptosis, and necroptosis, ferroptosis is a form of cell death that depends on iron-dependent lipid peroxidation^13^. The identification of small molecules that induce a nonapoptotic form of cell death led to the discovery of ferroptosis, and the term ferroptosis was coined by Brent Stockwell in 2012^47^. The major research areas that have provided the foundation for understanding ferroptosis are a) iron homeostasis, b) reactive oxygen species (ROS) biology, and c) amino acid and lipid metabolism, which are very much interconnected for inducing ferroptosis (Fig. 4). The oxidized form of cysteine, an amino acid that is required for the survival and growth of certain cells, causes glutathione (GSH) depletion and cell death^48^. GSH blocks the ability of oxidants such as hydrogen peroxide to cause oxidative stress. An antiporter Xc^–^, which imports cystine and is a building block for GSH, normally functions as a strong suppressor of ferroptosis^47^. A selenocystine protein, glutathione peroxidase 4 (GPX4), functions as a GSH-dependent peroxidase to prevent lipid oxidation in membranes^49^. PUFAs need to be incorporated into membrane lipids, such as phospholipids, which are key building blocks of membranes, to serve as essential substrates for ferroptosis. The enzymes involved in activating and incorporating polyunsaturated fatty acids (PUFAs) into membrane lipids promote ferroptosis. A mutagenesis screen in the KBM7 cell line, an analysis of ferroptosis-resistant cell lines, and a CRISPR suppression screen have revealed inactivation of acylcoenzyme A synthetase long-chain family member 4 and lysophosphatidylcholine acyltransferase 3 as a key mechanism for inhibiting ferroptosis in various contexts^47^. Consistent with the conclusion that oxidized PUFA tails must be associated with phospholipids to execute ferroptosis, the phospholipase A2 group VI suppresses ferroptosis by dissociating oxidized PUFA tails from phospholipids^50^. Therefore, free PUFAs or oxidized PUFAs are not intrinsically toxic to cells.

Identified in a high-throughput screen for HRAS^V12^-selective lethal molecules, erastin (eradicator of RAS-transformed cells) and RSL3 (RAS-selective-lethal-3) induce ferroptosis by inhibiting cystine uptake through system X_c_^–^ and by inhibiting GPX4, respectively^51,52^. GPX4 degradation by ferroptosis-inducer-56 (FIN56), identified in a screen for caspase-independent lethal compounds, induces ferroptosis^53^. FIN56 also increases sensitivity to ferroptosis by depleting coenzyme Q10 (CoQ10). However, ferroptosis suppressor protein 1 inhibits ferroptosis independent of GPX4 by regenerating the reduced form of CoQ10^13^.

## Crosstalk among RCD pathways

Although initially identified as independent pathways, pyroptosis, apoptosis, and necroptosis show extensive interactions among each other. The molecules that are involved in pyroptosis regulate apoptosis and vice versa. Activation of CASP7 observed in conditions known to induce pyroptosis, including *Salmonella* infection and LPS plus ATP, is abolished in macrophages deficient in CASP1^54,55^, demonstrating a regulatory role for CASP1 in CASP7 activation. However, macrophages deficient in GSDMD, a downstream molecule of CASP1 and executioner of pyroptosis, undergo apoptosis accompanied by CASP3 activation in response to inflammasome stimuli^56^. CASP1 activation reroutes cell death responses to GSDME-mediated secondary necrosis or pyroptosis via the Bid-CASP9-CASP3 axis in the absence of GSDMD^56^. It is possible that inflammasome-driven apoptosis might be physiologically relevant to cells with low or no GSDMD expression, such as neurons and mast cells. In addition, the triggers of NLRP1b and NLRC4 promote CASP8 activation in the absence of inflammasome components. In WT macrophages infected with *Salmonella*, CASP1 and CASP8 colocalize with ASC specks, although CASP8 is dispensable for *Salmonella*-induced pyroptosis^57^. However, ASC colocalizes with FADD in *Casp1*^–/–^ cells stimulated with the NLRC4 trigger FlaTox to promote Casp8-dependent apoptosis^58^. Furthermore, AIM2 inflammasome triggers, such as *Francisella* infection or DNA electroporation, lead to the recruitment and activation of CASP8 through ASC, resulting in CASP3 activation in *Casp1*^–/–^ cells^59,60^. In addition to its classical role in extrinsic apoptosis, CASP8 has been shown to regulate inflammasome activation and pyroptosis in various conditions. Inflammasome activation and cell death are reduced in macrophages lacking RIPK3 and CASP8 or RIPK3 and FADD compared with cells deficient in RIPK3 in response to LPS + ATP stimulation and *C. rodentium* or *Yersinia* infection, indicating that FADD and CASP8 regulate inflammasome activation and pyroptosis during NLRP3 inflammasome stimuli^61–63^. CASP8 promotes the priming and posttranslational modification of NLRP3, which are essential for activation of the NLRP3 inflammasome. In addition to its role in the upregulation of *Nlrp3* and *Il1b*, CASP8 is recruited into the inflammasome complex in response to NLRP3 triggers^61^. Furthermore, CASP8 can cleave GSDMD and activate the NLRP3 inflammasome upon TAK1 inhibition^64^. The crosstalk between pyroptosis and apoptosis has also been observed in conditions other than with inflammasome triggers. During chemotherapy, CASP3 cleaves GSDME to induce pyroptosis. CASP3 can also cleave GSDMD at its N-terminus to generate an inactive fragment that potentially limits GSDMD-mediated pyroptosis^38,65^. Bile acid-induced APAF1 apoptosomes containing CASP11 induce CASP3 cleavage to drive GSDME-dependent pyroptosis^66^. However, the regulatory mechanisms for switching between the APAF1-CASP11 pyroptosome and APAF1-CASP9 apoptosome remain elusive. Overall, these findings suggest that pyroptosis and apoptosis, which are functionally distinct cellular responses, mutually regulate each other.

There are few studies that suggest the interplay between pyroptosis and necroptosis. NLRP3 inflammasome activation occurs during necroptosis engaged by TLR3 signaling in combination with caspase inhibition. Cells deficient in RIPK3 or MLKL show impaired ASC oligomerization in response to Poly I:C and zVAD treatment, suggesting the involvement of RIPK3^67^. Potassium efflux through MLKL pores acts as a signal for NLRP3 inflammasome activation during cell death induced by necroptotic triggers^68^. The interactions between necroptosis and apoptosis have been comparatively well documented compared with those between necroptosis and pyroptosis. The balance between necroptosis and apoptosis is crucial to maintain homeostasis. While its activation triggers apoptosis, deletion of CASP8 in mice leads to embryonic lethality, which can be rescued by loss of RIPK3 or MLKL^69,70^^,^ suggesting a predominant role of apoptotic CASP8 in preventing necroptosis during development. Since RIPK1 is involved in the regulation of both necroptosis and apoptosis, embryonic lethality in *Ripk1*^–/–^ mice can only be rescued upon deletion of both CASP8 and RIPK3^71^. However, there are very limited studies on the intersection of ferroptosis with other RCD pathways. Nec-1, which blocks RIPK-1-dependent necroptosis, has been reported to inhibit erastin- or sulfasalazine-induced ferroptosis in Huh7 and SK-HEP-1 cells^72^. The inhibitory function of Nec-1 in ferroptosis could be an off-target effect on the ferroptosis pathway.

Overall, RCD processes such as pyroptosis, apoptosis, and necroptosis were originally thought of as distinct pathways. Emerging studies have suggested the existence of multiple interactions among these RCD pathways.

## PANoptosis

As described above, the crosstalk among pyroptosis, apoptosis, and necroptosis indicates the existence of a dynamic molecular interaction network that has conceptualized PANoptosis as an inflammatory RCD activated by specific triggers and with the molecular characteristics of pyroptosis, apoptosis, and necroptosis^73^ (Fig. 5). A study that showed the activation of CASP1, CASP8, and CASP3 and phosphorylation of MLKL, the key molecular events of pyroptosis, apoptosis, and necroptosis, in macrophages infected with influenza A virus (IAV) was the first to provide evidence for PANoptosis^74^. Cells lacking individual components of the typical RCD pathways show a similar degree of cell death compared to that in WT cells during IAV infection. However, loss of the ZBP1 or Zα domain of ZBP1 provides protection against cell death, indicating that the sensing of IAV occurs through its Zα domain^74–76^. ZBP1 initiates the activation of the molecular machinery for PANoptosis execution. Another cytosolic sensor, AIM2, induces PANoptosis in macrophages upon infection with HSV1 or *F. novicida*. While loss of ZBP1 or pyrin reduces cell death, combined loss of ZBP1 and AIM2 abrogates cell death and activation of PANoptosis, indicating that ZBP1 and pyrin regulate AIM2 responses during HSV1 and *F. novicida* infection^22^. Moreover, the PAN apoptotic cell death in macrophages infected with murine hepatitis virus (MHV), a beta coronavirus, is potentiated by ZBP1 upon IFN treatment^19^. In addition to viral and bacterial infection, ZBP1 also mediates PANoptosis during fungal infection^77^. NAIP-NLRC4-engaged bacteria, *Salmonella* and *Pseudomonas*, and TAK1-inhibiting bacteria, *Yersinia*, have been shown to induce PANoptosis^78,79^. In addition to pathogens, sterile triggers induce PANoptosis in macrophages^18,21,80^. Accumulation of dsRNA in cells stimulated with IFN plus NEI, such as KPT or leptomycin, is sensed by the Zα domain of ZBP1 to drive RIPK3- and CASP8-dependent PANoptosis^21,80^. The combination of TNF and IFN-γ activates STAT1, induces IRF1-dependent iNOS and subsequently produces NO, which triggers CASP8-dependent PANoptosis in macrophages^18,80^. However, human cancer cells undergo PANoptosis in an IRF1-dependent but NO-independent manner^23^, suggesting that there may be cell type- or species-specific differences in regulation. PANoptosis has also been demonstrated in sterile inflammation. Mice carrying a mutation in *Pstpip2* (*Pstpip2*^cmo^) develop osteomyelitis. Combined deletion of molecules involved in pyroptosis, apoptosis, and necroptosis can rescue these mice, implicating PANoptosis in this process^81,82^. Similarly, the expression of enzymatically inactive CASP8 (*Casp8*^*C362S/C362S*^) causes embryonic lethality in mice by inducing necroptosis and pyroptosis. However, *Casp8*^*C362S/C362S*^*Mlkl*^*–/–*^*Casp1*^*–/–*^ mice are viable^83,84^^,^ suggesting that the enzymatic activity of caspase-8 controls PANoptosis. Overall, distinctive upstream triggers or sensors mostly converge on CASP8, which functions as a central node for the execution of PANoptosis.

## Physiological relevance of cell death

Depending on the context, RCD pathways can be beneficial or detrimental. RCD pathways have mostly been implicated in combating infections by removing the replicative abilities of pathogens and preventing cancer by inducing cancer cell death. Conversely, aberrant cell death contributes to cytokine storm-associated and chronic degenerative diseases.

## Development and homeostasis

Among the various RCD pathways, apoptosis is best characterized for its role in normal animal development and tissue homeostasis. Targeted disruption of caspases in mice has revealed differential requirements for individual caspases during mammalian development. Caspase-8 deficiency leads to embryonic lethality, which is associated with a regression of the extraembryonic yolk sac vasculature followed by abdominal hemorrhage due to cardiac puncture^85^. The conditional deletion of caspase-8 in endothelial cells results in embryonic lethal phenotypes similar to global caspase-8-deficient conditions^86^. Although mice lacking caspase-8 in T cells or myeloid cells are viable, these mice appear to have deficiencies in expansion and activation of T-cells and differentiation of myeloid progenitors into macrophages^87^. Deletion of the necroptotic effector molecules RIPK3 or MLKL rescues the embryonic lethality of *Casp8*^–/–^ mice, suggesting that CASP8 prevents necroptosis during development^88^. Mice lacking RIPK1 die postnatally due to massive necroptosis in epidermal cells and apoptosis in the intestine^89^. Combined loss of CASP8 and RIPK3 in *Ripk1*^–/–^ mice rescues lethality^71^. Mice carrying an uncleavable RIPK1 (D325A) also undergo embryonic lethality, which can be rescued by combined loss of both apoptosis and necroptosis. CASP8 cleaves RIPK1 to prevent excessive necroptosis and apoptosis^90^.

Caspase-9 deficiency also results in embryonic lethality due to severe brain malformation and hindbrain neural tube defects. The phenotypes observed in *Casp9*^–/–^ mice are associated with reduced neuronal apoptosis, accumulation of necrotic tissues in the brain, and frequent intracerebral hemorrhages, highlighting the importance of CASP9 in brain development^91^. Caspase-3-deficient 129/Sv mice also show similar phenotypes as *Casp9*^–/–^ mice^92^. These findings confirm that caspase-3 activation occurring downstream of caspase-9, which has been suggested from in vitro studies, is essential in mediating neural cell death that is required for brain development. Notably, *Casp3*^–/–^ C57Bl/6 mice are normal, suggesting the possible presence of strain-specific genes that can actively suppress the phenotype caused by caspase-3 loss. Loss-of-function mutations in CASP9, APAF1, and CASP3 have been associated with neural tube defects^93^.

ADAR1- and ZBP1-regulated PANoptosis has been implicated in development and survival. Embryonic lethality in *Adar1*^–/–^, *Adar1*^p150null/p150null^, and *Adar1*^E861A/E861A^ mice, which have a point mutation in the ADAR1 catalytic domain, is accompanied by hyperproduction of type I IFNs, upregulation of ISGs, and widespread cell death, particularly in liver hematopoietic cells^80,94–96^. The loss of MDA5 or the downstream adaptor protein MAVS rescues the embryonic lethality of *Adar1*^–/–^ and *Adar1*^p150null/p150null^ mice, although these mice still undergo lethality shortly after birth. With the concurrent loss of ZBP1, survival is significantly improved in *Adar1*^–/–^*Mavs*^–/–^ mice or *Adar1*^p150null/p150null^*Mavs*^–/–^mice^94–96^, suggesting that developmental lethality is mediated by simultaneous activation of ZBP1, MDA5, and potentially other pathways. Beyond ADAR1-deficient conditions, ZBP1 also contributes to other developmental defects. The role of ZBP1 in driving lethality has further been shown in *Adar1*^P195A/p150null^ mice, which carry a mutation within the Zα domain (P195A) on one allele in combination with deletion of ADAR1^p150^ in the second allele of *Adar1*, and in *Adar1*^Zα/–^ mice^95^. Mice expressing RIPK1 with a mutated RHIM domain (mRHIM) undergo perinatal lethality, which can be rescued by deletion of ZBP1^97^ or mutation of the ZBP1 Zα domain^75^.

## Infectious diseases

All RCD pathways have been implicated in infectious diseases. Depending on the type of infection, these RCD pathways can be detrimental or beneficial. Lytic cell death pathways such as pyroptosis, necroptosis, ferroptosis, and PANoptosis are important for clearing invading pathogens. Pyroptosis releases intracellular bacteria residing within macrophages, such as *Burkholderia thailandensis*, *Salmonella* Typhimurium, and *Legionella pneumophila*, which are subsequently killed by neutrophils via a mechanism dependent on the production of ROS^98^. However, neutrophils in *Nox2*^–/–^ mice infected with *Pseudomonas aeruginosa* undergo pyroptosis to compensate for deficiency of another major antimicrobial pathway^99^. The physical extrusion of infected enterocytes from the intestine depends on NLRC4 inflammasome activation during *Salmonella* infection^100^. *Gsdmd*^–/–^ or *Il18*^–/–^ mice are more susceptible to *Salmonella* infection, suggesting that pyroptosis-mediated cytokine release promotes host protection against *Salmonella* infection^101^. GSDMD can also damage and lyse bacteria directly by binding to cardiolipin and oligomerizing to form pores on the bacterial cell membrane^102,103^. HIV infection induces pyroptosis in quiescent lymphoid CD4 T cells, consequently leading to CD4 T-cell depletion and chronic inflammation^104^.

Many bacteria and viruses are known to induce necroptosis by activating RIPK3 and MLKL. Most studies have shown that necroptosis has a detrimental effect on bacterial infection. Necroptosis induced by *Staphylococcus aureus* leads to tissue damage and mortality^105^. Pore-forming toxins produced by various bacteria, such as *Streptococcus marcescens*, *L. monocytes*, *S. aureus*, and *S. pneumonia*, induce necroptosis to promote acute bacterial pneumonia^106^. Administration of RIPK1 or MLKL inhibitors has been shown to reduce morbidity and mortality during *S. marcescens* hemorrhagic pneumonia^106^. In contrast, necroptosis signaling is essential for host defense against *S. pneumonia*. Higher plasma concentrations of RIPK3 could serve as a potential marker of pneumococcal pneumonia^107^. EspL, which is an effector of the type III secretion system of enteropathogenic *E. coli*, degrades the RHIM-containing proteins RIPK1, RIPK3, TRIF, and ZBP1 to restrict pyroptosis, apoptosis and necroptosis during infection^108^. *E. coli* expressing NleB1 inhibits apoptosis and necroptosis by modifying arginine residues in death domain-containing proteins such as FADD and RIPK1^109,110^. NleB1-deficient *E. coli* fails to colonize the intestine of the host, suggesting the protective role of apoptosis and necroptosis during *E. coli* infection. The secreted effector of *Toxoplasma gondii*, TgNSM, prevents host cell necroptosis, thereby assuring the survival of intracellular cysts leading to chronic infection^111^. In addition, RIPK3 deficiency in combination with caspase-8 or FADD leads to increased susceptibility to *Yersinia* infection^62^.

Pathogens regulate ferroptosis by influencing host iron metabolism, iron transport, ROS production and antioxidant defenses^13^. Ferroptosis restricts hepatitis C viral replication in the host cell^112^. Patients with COVID-19 have high serum ferritin levels, indicating high iron exposure in tissues. Although the ferroptosis marker TfR1 is induced in Syrian golden hamsters following SARS-CoV-2 infection^113^, the significance of ferroptosis in SARS-CoV-2 infection and COVID-19 remains unclear. *Mycobacterium* and *Pseudomonas* infection induce ferroptosis, which upon inhibition increases the infection, indicating that ferroptosis promotes *Mycobacterium* and *Pseudomonas* infection^114,115^.

The role of PANoptosis has been well studied during IAV and SARS-CoV-2 infection^19,74,116,117^. IAV infection induces NLRP3 inflammasome activation and PANoptosis in macrophages, which is dependent on the Zα domain of ZBP1^74,75^. The mortality caused by IAV infection in WT mice is prevented upon loss of the ZBP1 or Zα domain of ZBP1^19,118^, suggesting that ZBP1-mediated PANoptosis provides host defense against IAV infection. In contrast to IAV infection, PANoptosis contributes to worse outcomes in COVID-19. SARS-CoV-2 infection-induced mortality is reduced in mice upon administration of neutralizing antibodies against the PANoptosis activating cytokine combination TNF and IFN-γ^18^. The synergism of TNF and IFN-γ induces NO via the STAT1/IRF1 axis to activate PANoptosis in a caspase-8-dependent manner. Indeed, deletion of *Nos2* or *Casp8* reduces SARS-CoV-2 infection-driven weight loss without impacting peak viral burdens in mice^119^, indicating the pathogenic role of the iNOS-caspase-8 axis in COVID-19. Moreover, treatment with IFNs potentiates ZBP1-dependent PANoptosis during β-coronavirus infection, including MHV and SARS-CoV-2^19^. As a result of its antiviral properties, IFN therapy has been suggested to treat patients with viral infection. However, clinical studies have shown worse outcomes in patients with COVID-19 following IFN therapy^116^. Similarly, MHV-infected mice show increased mortality after administration of IFN-β. However, *Zbp1*^–/–^ mice show reduced PANoptosis and mortality following IFN-β administration compared with WT mice^19^, suggesting that ZBP1-mediated PANoptosis impedes the therapeutic efficacy of IFN-β in COVID-19. Furthermore, murine BMDMs and human THP-1 macrophages undergo AIM2-dependent PANoptosis during infection with HSV1 or *Francisella novicida*. Confocal microscopy shows colocalization of ASC with CASP8 and RIPK3, and immunoprecipitation studies have found interactions of ASC with CASP1, CASP8, RIPK3, AIM2, and Pyrin in macrophages infected with HSV1^22^.

## Tumorigenesis

The role of pyroptosis, apoptosis, necroptosis, and ferroptosis in cancer remains controversial because they can be both tumor suppressive and tumor promoter depending on the context^40,120^. For instance, in an AOM-DSS model of colorectal tumorigenesis, *Gsdme*^–/–^ mice show reduced tumor burden and HMGB1 release in the colon compared to their WT littermates^121^, implying that GSDME-mediated pyroptosis promotes colorectal tumorigenesis by releasing HMGB1. However, WT and *GSDME*-depleted cancer cells develop tumors of similar size in xenograft models of colorectal cancer, lung cancer, and melanoma^122^. Similarly, the role of necroptosis in tumorigenesis also seems to be controversial. The downregulation of RIPK3 or MLKL is associated with poor prognosis in patients with various types of cancer, such as colorectal cancer, acute myeloid leukemia, melanoma, breast cancer, ovarian cancer, and gastric cancer. On the other hand, the upregulation of RIPK3 or RIPK1 is associated with a promising prognosis in patients with lung cancer, glioma, and pancreatic cancer^120^. Blockade of the necrosome in vitro promotes proliferation of cancer cells and induces an aggressive oncogenic phenotype. Deletion of RIPK3 or blockade of RIPK1 has shown protection against pancreatic oncogenesis^123^, indicating that necroptosis promotes pancreatic tumorigenesis. Necroptosis can induce cancer cell death, leading to a promising prognosis, while it can also lead to inflammation and cancer.

Loss of apoptosis allows cancer cells to survive longer, resulting in the accumulation of mutations associated with different stages of tumorigenesis, such as proliferation, invasion, migration, and metastasis. There are several apoptosis-inducing anticancer drugs; however, cancer cells develop resistance to these agents^124^. Some agents, such as the combination of IFNs with nuclear export inhibitors or TNF and IFN-γ, have shown promising anticancer effects^21,23^. The combination of IFN with KPT induces ZBP1-dependent PANoptosis, which regresses melanoma growth in mice. PANoptosis induced by IFN plus KPT involves ADAR1, the only other human protein to contain a Zα domain. The interaction of ZBP1 with ADAR1 inhibits cell death, while its interaction with RIPK3 promotes cell death^21^. Similarly, ZBP1-dependent cell death can improve responsiveness to immune checkpoint blockade therapy in mouse models of melanoma^125^. Furthermore, PANoptosis-associated genes, including ZBP1, have been associated with a better prognosis in patients with skin cutaneous melanoma. TNF administration plus IFN-γ-induced PANoptosis has shown preclinical promise in promoting cell death in human cancer cells to reduce tumor size in murine ectopic transplant models^23^. In addition, IRF1-dependent PANoptosis inhibits colorectal tumorigenesis^126^. Overall, the role of pyroptosis and necroptosis in tumorigenesis is controversial. However, agents that induce PANoptosis are promising in treating cancer.

## Autoimmune and inflammatory diseases

Deletion of GSDMD in *Mefv*^V726A/V726A^, a mouse model of familial Mediterranean fever (FMF), an autoimmune disease driven by mutations in the gene *Mefv*, leads to normal growth and rescues inflammation^127^. In a mouse model of alcoholic hepatitis, loss of GSDMD mitigates the development of steatohepatitis^128^, indicating that pyroptosis contributes to sterile inflammation in liver disease. Gain-of-function mutations in NLRP3 have been associated with cryopyrin-associated periodic syndromes (CAPS)^129^. As GSDMD is activated downstream of NLRP3, it is possible that pyroptosis is involved in CAPS. Indeed, loss of GSDMD ameliorated inflammatory symptoms in a mouse model of NLRP3 gain-of-function mutations^130^.

Deficiency of RIPK3 or MLKL prevents airway inflammation in mice subjected to cigarette smoke-induced experimental chronic obstructive pulmonary disease (COPD)^131^, suggesting that necroptosis signaling contributes to inflammatory responses, airway remodeling and emphysema in COPD. The lungs of patients with COPD display active RIPK3 and MLKL. Administration of Nec-1 or loss of RIPK3 protects the liver from alcoholic and drug-induced liver injury^132,133^.

Ferroptosis has been associated with systemic lupus erythematosus (SLE). Patients with SLE and mice prone to lupus show low neutrophil counts and increased lipid ROS production. Neutrophil-specific *Gpx4* haplosufficiency mirrors key clinical features of human SLE, including autoantibodies, neutropenia, skin lesions and proteinuria, suggesting that neutrophil ferroptosis leads to neutropenia and disease manifestations in SLE^134^. Indeed, administration of a ferroptosis inhibitor ameliorated disease severity in lupus-prone mice. Moreover, autoantibodies and IFN-α induce ferroptosis in neutrophils by suppressing Gpx4 expression^134^. Thus, ferroptosis in neutrophils promotes autoimmune disease, possibly by releasing autoantigens. Similarly, ferroptosis in human airway epithelial cells is associated with the release of mitochondrial DNA and consequent worse asthma patient outcomes^135^.

Several preclinical studies have indicated the role of PANoptosis in inflammatory diseases. Mutation of the proline-serine-threonine phosphatase-interacting protein 2 gene in mice (*Pstpip2*^cmo^) causes inflammatory lesions in the bones and various degrees of skin and paw inflammation, closely resembling the human disorder known as chronic recurrent multifocal osteomyelitis. While neutrophils and IL-1β are critical in the initiation of the disease, the combined deletion of RIPK3, caspase-1, and caspase-8 – all critical components of PANoptosis – prevents cytokine release and disease progression in *Pstpip2*^cmo^ mice^81^. Induction of PANoptosis by proinflammatory cytokines, particularly the synergism of TNF and IFN-γ, contributes to lethal shock in a mouse model, which mirrors the major symptoms of cytokine storm associated diseases. The mortality driven by PANoptosis is prevented upon loss of STAT1 or codeletion of CASP8 and RIPK3^18^. ZBP1-mediated PANoptosis contributes to the pathology of several diseases. In humans, ADAR1 loss-of-function or MDA5 gain-of-function mutations have been identified in rare autoimmune diseases such as AGS. Furthermore, mutations in the ADAR1 Zα domain cause AGS and BSN when combined with alleles that cause loss of ADAR1^p150^ expression. These conditions are mimicked in *Adar1*^mZα/–^ or *Adar1*^P195A/p150null^ mice, and these phenotypes are rescued by concomitant deletion of ZBP1 or the ZBP1 Zα domain^94,95^. Additionally, mice lacking SETDB1 in intestinal epithelial cells have severe bowel inflammation, which is prevented by deletion of ZBP1^136^. These findings have established a possible pathological role of ZBP1-dependent PANoptosis in common inflammatory diseases.

## Summary

Innate immunity-mediated cell death plays critical roles across homeostasis, development, autoinflammatory diseases, host defense, and tumorigenesis. While nonlytic cell death–apoptosis is mostly implicated in homeostasis and development, lytic cell death pathways such as pyroptosis, necroptosis, ferroptosis, and PANoptosis are associated with infectious and autoinflammatory diseases. The RCD pathways discussed in this review are mostly distinguished by molecules, morphologies, and stimuli. Among the lytic cell death pathways, pyroptosis and PANoptosis are generally associated with the release of IL-1β and IL-18. Since cancer cells develop resistance to therapeutics, switching a mode of cell death to another or engaging more than one type of RCD could be beneficial. Importantly, agents that regulate PANoptosis show great potential for the treatment of cancer.

These RCD pathways share molecules that execute cell death. Depending on the complex assembled by the shared molecules, cells commit to one of these RCD pathways. For instance, inflammasomes execute pyroptosis, apoptosomes and complex II drive intrinsic and extrinsic apoptosis, necrosomes result in necroptosis, and PANoptosomes execute PANoptosis. The assembly of these complexes depends on the type of stimulus. However, the complex responsible for the execution of ferroptosis is not clear. While pyroptosis and necroptosis are accompanied by plasma membrane rupture mediated by NINJ1 for the release of larger DAMPs^14^, the role of NINJ1 in ferroptosis and PANoptosis has not been studied. While our understanding of the fundamental role of RCD pathways has improved, many questions remain. For instance, how does a dedicated molecule of an RCD pathway influence another RCD pathway? What is the identity of additional sensors and triggers? These RCD pathways also require further molecular characterization in different cell and tissue types. Since RCD pathways and their molecular components have been widely implicated across the disease spectrum, continued research on these RCD pathways is needed to discover therapeutic targets.
